# Identification of Autologous Tissue Sources and Optimization of a Treatment Method for Intraoperative Manufacturing of Heart Valve Constructs

**DOI:** 10.1111/aor.70151

**Published:** 2026-04-26

**Authors:** Marvin Steitz, Mahamuda Badhon Khan, Katharina Fritsch, Alexander Breitenstein‐Attach, Jana Katharina Betty Korbacher, Jordi Joaquin Modolell Y Mainou, Katrin Schrimpf, Robert Ramm, Sara Knigge, Katja Eildermann, Georg Arlt, Antonia Schulz, Jonathan Frederik Sebastian Kiekenap, Boris Warnack, Peter Kramer, Frank Edelmann, Felix Berger, Boris Schmitt

**Affiliations:** ^1^ Department of Congenital Heart Disease—Pediatric Cardiology Deutsches Herzzentrum der Charité Berlin Germany; ^2^ Charité—Universitätsmedizin Berlin, Corporate Member of Freie Universität Berlin Humboldt‐Universität zu Berlin Berlin Germany; ^3^ DZHK (German Centre for Cardiovascular Research) Partner Site Berlin Berlin Germany; ^4^ Department Dynamics and Transport in Quantum Materials Helmholtz‐Zentrum Berlin für Materialien und Energie GmbH Berlin Germany; ^5^ Lower Saxony Centre for Biomedical Engineering, Implant Research and Development University of Veterinary Medicine Hanover Hannover Germany; ^6^ Department of Pediatric Cardiology and Intensive Care Medicine Georg‐August University Göttingen Göttingen Germany; ^7^ Department of General Surgery, Minimally Invasive and Visceral Surgery, Selected Vascular Surgery Park‐Klinik Weißensee Berlin Germany; ^8^ Warnack Medconsult Untere Wenkenhofstrasse Riehen Switzerland

**Keywords:** autologous, biomaterials, cardiology, pericardium, peritoneum

## Abstract

**Introduction:**

Valvular heart disease remains a leading cause of morbidity and mortality. Current biological and mechanical heart valve prostheses have significant limitations and fail to address the patient's needs. Most of the limitations arise from the use of foreign materials, which can largely be addressed by utilizing autologous pericardial tissue. However, not all patients have usable pericardium due to previous interventions. This study investigates peritoneum as a potential alternative to pericardium and evaluates an optimized treatment method for intraoperative tissue treatment.

**Methods:**

A previously established cross‐linking method was optimized and applied to pericardial and peritoneal tissues. The tissues were characterized with respect to biochemical composition, cross‐linking degree, biomechanical properties, and structural organization. Furthermore, the feasibility of valve shaping was assessed, and constructs were subjected to acute hydrodynamic testing.

**Results:**

Tissue treated with the optimized cross‐linking method showed high cytocompatibility and was successfully applied to peritoneal tissue. Compared to pericardium, peritoneum exhibited a higher elastin content and a looser structural organization, resulting in distinct mechanical behavior. Both tissues could be shaped into valve constructs showing adequate acute hydrodynamic behavior under the applied test conditions.

**Conclusion:**

The findings suggest that the peritoneum is a promising candidate material for further investigation as a heart valve scaffold. However, differences in microstructure and mechanical behavior highlight the need for further testing, particularly regarding long‐term durability, cyclic loading behavior, and tissue remodeling capacity.

## Graphical abstract

The use of autologous connective tissue, such as pericardium or peritoneum, combined with controlled tissue treatment, has the potential to result in a more durable heart valve prosthesis than current commercial prostheses.

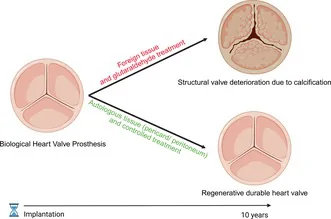

**Abbreviations**
coll‐e: collagen Young's modulus
DSC: differential scanning calorimetry
ECM: extracellular matrix
EOA: effective orifice area
GLUT: glutaraldehyde
HUVEC: human umbilical vein endothelial cell
PBS: phosphate‐buffered saline
RF: regurgitation fraction
SHG: second harmonic generation
SMA: smooth muscle actin
*T*_d_
: denaturation temperature
*T*_onset_
: endothermic onset temperature
UTS: ultimate tensile strength
XOP: phenolic crosslinking agent

## 1 Introduction

By opening and closing, heart valves allow the unidirectional flow of blood during the cardiac cycle, whereas preventing the backflow of blood [1]. Each heart valve opens and closes about 103 000 times a day and is subjected to a large amount of flexion for billions of cycles in its lifetime. Until today, no man‐made structure can meet this achievement. Various pathological conditions can cause heart valve malfunctions, leading to severe disorders [2]. Since defective or diseased heart valves are unable to recover, patients face severe risks of progressive heart failure and death. Pediatric patients face an even more challenging prognosis since they also require re‐operations due to the outgrowth of their prostheses. This adversely impacts cardiac and overall physiological development and quality of life [3]. Currently, the only treatment option for heart valve dysfunctions is surgical or interventional replacement, of which 200 000 are conducted annually worldwide [4]. Additionally, 1% of all newborns suffer from congenital heart disease, of which 30% concern the heart valves [5].

Commercially available heart valve prostheses are made from either artificial materials (mechanical valves) or biological materials from foreign sources (xeno‐ or homo‐grafts). Although both types show benefits and drawbacks for adult patients, there is no heart valve prosthesis available that meets the needs of pediatric patients. Mechanical heart valves are durable yet lack a physiological geometry and have a foreign surface, leading to high shear stresses and jetstreams that cause damage to red blood cells. The foreign surface of mechanical valves also increases platelet activation, making thrombogenicity their most prevalent complication, necessitating the life‐long use of anticoagulants [3]. Polymeric heart valves, none of which is commercially available yet [6], can be fabricated from various materials by casting or electrospinning. The most common materials include polyurethane, polytetrafluoroethylene, biodegradable elastomers, hydrogels, coated meshes, and nanofibers. However, until today, all of them show poor durability and dynamic mechanical behavior [7]. Bioprostheses, on the other hand, demonstrate excellent hemodynamic properties but are prone to structural valve degeneration, which usually occurs within 7–15 years. Until today, the host immune response cannot be completely suppressed, which leads to foreign body reactions. These can vary greatly between patients [8], for example, calcification of bioprostheses is accelerated in children [9].

None of the current prostheses fully meets the requirements for an ideal heart valve formulated by Harken [10] or Fioretta et al. [3], who stated that a prosthesis should be capable of self‐repair, adaptively remodeling, and growth, continuously adjust to functional and somatic changes, and offer a durable solution for all patients. Furthermore, the prosthesis should not degenerate, be immunogenic, or necessitate lifelong antithrombotic therapy. Current approaches of tissue‐engineered heart valves show promising first approaches in meeting these criteria, but their clinical relevance remains non‐existent due to inappropriate architecture, immune‐mediated degeneration, incomplete recellularization or decellularization, and graft failure at systemic pressures, resulting in deformation under stress [3, 11].

In the field of heart valve therapy, materials that may enable improved biocompatibility and potential for adaptation remain an unmet need [3].

Since the use of viable or recellularizable tissue is essential for a heart valve with self‐regulation properties [12, 13], autologous tissue is an excellent source for producing such a prosthesis. To date, the Ross procedure, in which an untreated autotransplant is utilized, remains the only heart valve replacement technique capable of regeneration and remodeling [3, 8]. It demonstrates immune compatibility, the potential for somatic growth adaptation, a low risk of thrombosis, and superior hemodynamic performance. As a result, it continues to be the most widely used surgical method in pediatric patients [14, 15]. However, it transforms a “one‐valve disease” into a “two‐valve disease” [15] and shows a high early mortality rate [11].

These limitations could be overcome by the conversion of autologous tissue. The Ozaki Procedure demonstrates the proof‐of‐concept for converting autologous tissue into a heart valve prosthesis [16]. Therefore, a small piece of the patient's pericardium gets excised and reconstructed into a new valve [17]. Due to the utilization of autologous tissue, the Ozaki‐Procedure shows less infectivity and excellent biocompatibility [18]. However, the Ozaki procedure is a complex surgical procedure [12], utilizing glutaraldehyde treated tissue.

To combine the advantages of both approaches, we propose utilizing tissue of autologous origin, which gets 3‐dimensional reshaped into a heart valve and treated without glutaraldehyde. Such tissue may retain aspects of its native biological properties [13]. Although pericardium is a promising tissue, internal data indicate that many surgically pre‐operated patients often have damaged pericardial tissue, making it unsuitable for heart valve production. In addition, the extraction of pericardial tissue is associated with various risks. Other collagen‐based autologous tissues for reconstructing a heart valve have been used before (e.g., fascia lata) [19, 20]. However, none have demonstrated results comparable to those of a native heart valve or one reconstructed from pericardial tissue, highlighting the need to identify an alternative source.

This study aims to identify an alternative tissue to pericardium and to optimize a corresponding treatment method for the intraoperative manufacturing of a heart valve construct. We chose peritoneum as the assessed alternative tissue, as it can be easily removed without causing consequential harm to the patient and is already used in the manufacturing of bioprostheses such as in visceral surgery to create vascular substitutes for the portal vein. Furthermore, its collagen‐based nature allows for controllable stability via cross‐linking, making it a candidate for further transformation and evaluation [2].

Since a tissue's (physical) properties are mainly determined by its cells, the extracellular matrix (ECM) components, and their relative quantities [18], we assessed peritoneum for its biochemical, biomechanical, and further properties with a focus on initial in vitro characterization and feasibility assessment, whereas comparing it to pericardium. Additionally, we optimized our cross‐linking method for the treatment of pericardial and peritoneal tissue.

The manufacturing of autologous pericardial valve constructs with encouraging in vivo performance has been demonstrated in previous studies [21]. In addition, the treatment method, which was further optimized in this study, was previously characterized and modified for an intra‐operative application [22].

## 2 Materials and Methods

All tests were performed in replicates (*n* ≥ 3) utilizing porcine tissue. Pericardial tissue (treated and untreated) served as the control group. Where possible, human tissue was examined; however, replicates could not always be ensured due to limited availability. Ethical approval (EA2/104/23) was obtained from the Ethics Commission of the Charité—Universitätsmedizin Berlin for using human tissue. Unless otherwise stated, the tissue is understood to be of porcine origin.

### 2.1 Processing of Tissue

An abattoir provided the tissue used to optimize the treatment method (Duroc Breed; Fleischerei Lehmann, Trebbin, Germany).

To compare peritoneum as an alternative to pericardium, porcine tissue was obtained from cardio pigs (Heinrichs Forschungsschweine, Aachen, Germany) from the Research Institutes for Experimental Medicine (Charité—Universitätsmedizin Berlin, Berlin, Germany) following the euthanization of the animals. Although the porcine pericardium was excised above the left ventricle as described elsewhere [23], the peritoneal tissue was obtained from the anterolateral abdominal region at the transition to the rectus fascia. Human tissue (pericardium and peritoneum) was obtained from respective surgical departments (Charité—Universitätsmedizin Berlin, Berlin, Germany).

### 2.2 Tissue Treatment

Transportation, cleaning, and storage of the tissues were performed as described before [22]. Prior to the assessments, the tissue was either treated with a phenolic crosslinking agent (XOP), glutaraldehyde (GLUT) (Thermo Fisher Scientific, Waltham, MA, USA) dissolved in phosphate‐buffered saline (PBS) (Thermo Fisher Scientific, Waltham, MA, USA), or left untreated, as shown in Table 1. Based on previous results, the former‐identified XOP concentration of 1.5% or 2.0% [22] was further diluted to 0.4%.

**TABLE 1:** Utilized treatment methods.

Treatment	Concentration & incubation time
GLUT	0.625% for 24 h, according to the commercial concentration [24]
XOP	0.4% for 1 h (adapted from former procedure [21])
Untreated	/

To simulate the conditions of the operating room and the intraoperative manufacturing process, the tissue was simply placed in the cross‐linking solution without using a shaker.

The cross‐linking reaction was terminated by rinsing the samples with PBS. Afterward, the samples were either processed immediately or stored at 4°C as described elsewhere [22].

For the selected treatment (0.4% XOP for 1 h), the principle “as much as necessary, as little as possible” was applied. Accordingly, the tissue should be altered as little as possible to preserve its self‐regulatory properties, whereas still being sufficiently cross‐linked to ensure adequate mechanical stability and protection against enzymatic degradation [25, 26].

### 2.3 Optimization of Tissue Treatment

#### 2.3.1 Biocompatibility Testing

For cytocompatibility testing of 0.4% XOP, a direct contact assay was conducted in vitro according to the applicable standard [27]. The tests were exclusively conducted on porcine pericardium. First, pericardial tissue (2.4 cm^2^/patch) was disinfected in an antibiotic cocktail comprising 50 mg/L vancomycin (Sigma‐Aldrich, Saint Louis, MO, USA), 8 mg/L gentamicin (Sigma‐Aldrich, Saint Louis, MO, USA), 240 mg/L cefoxitin (Sigma‐Aldrich, Saint Louis, MO, USA), and 25 mg/L amphotericin B (Thermo Fisher Scientific, Waltham, MA, USA) at 37°C for 24 h under agitation (200 rpm), as described elsewhere [28]. The tissue was washed with sterile PBS firstly for 30 min and secondly overnight. The disinfected patches were cut into 5 mm punches, incubated with XOP or GLUT (*n* = 3) for 1 and 24 h, and washed in PBS for 10 min. Human umbilical vein endothelial cells (HUVEC) (PromoCell, Heidelberg, Germany) were seeded at a density of 5 × 10^4^ cells with 500 μL culture medium per well for 24 h for cells to attach. The cross‐linked patches were placed on the cell monolayer for 24 and 72 h and further processed utilizing LIVE/DEAD staining (Thermo Fisher Scientific, Waltham, MA, USA) and MTS assay (Promega, WI, Madison, USA). The LIVE/DEAD staining was adapted for tissue staining by adding 10 μL of staining solution and 40 μL culture media to each well, followed by incubation for 15 min at room temperature, and two times washing for 15 min to remove background signals. Images were realized using a Nikon Eclipse Ti‐U (Nikon, Minato City, Tokyo, Japan) within three channels and 4× and 10× objectives for magnification. For the total amount of cells, the amount of living cells, and the amount of dead cells, white light, GFP 488/515 (494/517 nm), and RFP 570/602 (528/617 nm) were used, respectively. The MTS assay was conducted as per the manufacturer's protocol.

### 2.4 Comparison of Pericardium and Peritoneum

#### 2.4.1 Biochemical Analysis

Biochemical analysis, namely collagenase and elastase assays, was conducted for different purposes:

1. Transferability of the cross‐linking method to peritoneum by determining the amount of introduced cross‐links and assessment of the treatment's protection against enzymatic digestion (exclusively by collagenase assay tested on cross‐linked tissues).
2. Quantification of ECM components by measuring the weight loss of untreated tissue after enzymatic digestion.

##### 2.4.1.1 Collagenase Assay

1 × 1 cm pericardium and peritoneum patches (*n* = 3, except for human‐ or valvular tissue) were treated with XOP or GLUT or were left untreated. To determine the dry weight of the tissue samples, they underwent freeze‐drying with a lyophilizer (Labconco FreeZone 2.5, Kansas City, MO, USA) before being weighed. Subsequently, the patches were incubated for 48 h at 37°C, with each 2 mL comprising 150 U collagenase type VII (Sigma‐Aldrich, Saint Louis, MO, USA) as described elsewhere [29]. After the incubation, the tissue samples were again freeze‐dried and weighed. Finally, the weight loss [%] was calculated as described elsewhere [30]:

$$\begin{array}{c} \text{Weight loss ratio}\\ =\frac{\text{Weight before hydrolysis}-\text{Weight after hydrolysis}}{\text{Weight before hydrolysis}}\times 100 \end{array}$$

##### 2.4.1.2 Elastase Assay

1 × 1 cm pericardium and peritoneum patches (*n* = 3, except for human‐ or valvular tissue) were left untreated. To determine the dry weight of the tissue samples, they underwent freeze‐drying with a lyophilizer (Labconco FreeZone 2.5, Kansas City, MO, USA) before being weighed. Subsequently, the patches were incubated for 24 h at 37°C with each 1.5 mL comprising 5 U elastase (ROTH, Karlsruhe, Germany) as described elsewhere [29]. After the incubation, the tissue samples were again freeze‐dried and weighed. Finally, the weight loss [%] was calculated as described elsewhere [30].

#### 2.4.2 Thermoanalytic Testing

To confirm the transferability of the treatment method from pericardial tissue to peritoneum and to compare the physiological amount of cross‐links in the tissues, the cross‐linking degree of treated and untreated samples was assessed using differential scanning calorimetry (DSC). Loke and Khor [31] have described DSC as the prioritized method for the validation of the shrinkage temperature of tissue, and therefore for measuring the extent of crosslinking within a tissue. The treated group's endothermic onset temperature (*T*_onset_), representing the protein denaturation temperature (*T*_d_) [32], and therefore the cross‐linking degree, was assessed using a DSC 404 F1 Pegasus from Netzsch (Netzsch DSC 404 F1 Pegasus; NETZSCH Gerätebau GmbH, Selb, Germany). The samples (*n* ≥ 3; 1.5–6.0 mg) were cut using a 5 mm biopsy puncher (Kai Medical, Seki City, Gifu, Japan) and placed in aluminum crucibles (Mettler Toledo, Columbus, OH, USA), which were hermetically sealed using a pressure welding machine (NETZSCH‐Gerätebau GmbH, Selb, Germany). Using an empty crucible as a reference, measurements were conducted in air of N_2_/O_2_ (80/20) at a flow rate of 20 mL/min. Additionally, air with a flow rate of 50 mL/min was used as a protective atmosphere. Scans were performed in a temperature range of +10°C–120°C with a heating rate of approximately +10°C min^−1^. The method was verified by a pilot run and compared with previous results [22], confirming the *T*_d_ of the untreated pericardial group. Netzsch software Proteus 8.0 (Netzsch DSC 404 F1 Pegasus; NETZSCH Gerätebau GmbH, Selb, Germany) was used as evaluation software.

#### 2.4.3 Biomechanical Testing

Tensile testing was conducted using an Instron 3365 dual‐column Universal Testing System (Instron, Bucks, UK) as described elsewhere [33]. Due to similar tissue behavior in both directions, as described elsewhere, testing was exclusively conducted along the tissue's dominant collagen fiber orientation (circumferential) [34]. Therefore, the collagen fiber alignment was determined using a lightbox. Each sample was cut into stripes of 5 mm width and 40 mm length and fixed utilizing sandpaper into clamps. Afterward, the specimens (*n* ≥ 3, except human tissue) were tested via low‐force uniaxial tensile testing at 37°C in a temperature‐controlled water bath. The samples were first preconditioned 10 times under cyclic loading at 0.1 N, followed by loading to failure at a rate of 20 mm/min. The load (F, in N) and the elongation (Δl, in mm) were recorded and converted into engineering stress (*σ*, in MPa) and strain (*ε*, dimensionless). The collagen Young's modulus (coll‐e, in MPa), failure strain (%), and ultimate tensile strength (UTS) were calculated as biomechanical parameters and used to plot respective stress–strain curves.

#### 2.4.4 Histology

To further compare peritoneal and pericardial tissue, a histological assessment was conducted. Therefore, tissue samples were cut into 1 × 1 cm patches and incubated in 4.0% formalin (Avantor Inc., Radnor, PA, USA) for 24 h. Subsequently, the samples were embedded in paraffin and sliced into 3 μm microtome sections. Afterward, Movat Verhoeff (#12061; Morphisto GmbH, Offenbach, Germany) and mouse anti‐human smooth muscle actin (Dako, M0851) were applied to all specimens as described elsewhere [35]. The target structures and colors are described in Table 2.

**TABLE 2:** Dyes used for tissue staining.

Dye	Target structure	Color
Movat Verhoeff	Elastic and connective tissue fibers	Five‐color stain visualizes nuclei in blue‐black, muscle and cytoplasm in red, ground substance in blue/turquoise, collagen in yellow, cartilage in blue‐green/yellowish, elastic fibers in black, bone in yellow/red, and fibrin in bright red
Smooth muscle actin	Smooth muscle cells	Brown

#### 2.4.5 Fiber Alignment Assessment

A Nikon A1R + Multiphoton system (Nikon, Minato City, Tokyo, Japan) equipped with a 25 × 1.1 numerical aperture water‐dipping immersion objective was used to examine the fiber alignment of collagen and elastin within the tissues, as described before [22]. Equally sized patches (*n* = 3) were cut from each group, immersed in PBS, fixed with a harp, and placed under the microscope. Using second‐harmonic generation (SHG), the collagen fibers were excited at a wavelength of 820 nm and an emission of 410 nm. The elastin fibers were excited via fluorescence signals with an emission between 525 and 550 nm.

#### 2.4.6 Hydrodynamic Testing

To assess short‐term mechanical stability of pericardial and peritoneal heart valve constructs, hydrodynamic testing (*n* = 3) was performed.

The hydrodynamic testing was carried out using a commercially available ViVitro pulse duplicator system (ViVitro Labs Inc., Victoria, Canada). As described elsewhere [36], a suitable heart valve prosthesis must show respective hemodynamic performance, which is characterized by the following parameters:

- Low mean pressure difference: Indicates efficient blood flow with minimal resistance.
- Correct valve kinematics: Timely opening and closing of the valve leaflets.
- Minimal regurgitation: Low backflow through the valve, indicating effective closure.
- Sufficient effective orifice area: Reflects the ability of the valve to allow sufficient blood flow.

Therefore, the heart valve constructs were tested under right heart conditions. Normotensive pulmonary pressure settings were selected in accordance with DIN EN ISO 5840‐1:2021 [37]. These conditions include a right ventricular peak systolic pressure of 18–35 mmHg, a pulmonary artery end‐diastolic pressure of 8–15 mmHg, and a peak differential pressure across the closed pulmonary valve of 13–28 mmHg. The target hemodynamic parameters were a cardiac output of 5.0 L/min, a heart rate of 70 bpm, a mean arterial pressure of 20 mmHg, and a systolic duration of approximately 35% for the cardiac cycle. In accordance with the applicable standard, 10 consecutive cycles were measured for each valve, and the average of the effective orifice area (EOA) and the regurgitation fraction (RF) was calculated [37].

##### 2.4.6.1 Manufacturing of Heart Valve Constructs

Excised pericardial and peritoneal tissue patches (≥ 5 × 5 cm) were cleaned of adipose tissue and placed into the interlocking parts of a 3D‐printed mold (with a diameter of 22 mm), which shapes the tissue into a three‐dimensional heart valve construct. Afterward, the tissue was cross‐linked for 1 h utilizing 0.4% XOP and sutured into a balloon‐expandable Optimus‐XXL Stent (Andtratec, Koblenz, Germany). To separate the single leaflets, a self‐designed cutting tool was used. The stented heart valve constructs were then crimped onto an Altosa‐XL‐GEMINI PTA Balloon Catheter (Andtratec, Koblenz, Germany) utilizing an Edwards Crimper 9600CR (Edwards Lifesciences, Irvine, CA, USA). Finally, the heart valve construct was delivered in a minimally invasive manner to the landing zone (mock vessel).

### 2.5 Statistical Analysis

GraphPad Prism version 8.2.0 for Windows (GraphPad Software, San Diego, California, USA) was used for statistical analysis. If applicable, the single variables were shown individually or were expressed as the mean with a standard deviation. Group comparisons were conducted using one‐way ANOVA, with significance set at *p* ≤ 0.05.

## 3 Results

### 3.1 Optimization Tissue Treatment

#### 3.1.1 Biocompatibility Testing

To optimize the treatment method for manufacturing autologous heart valve constructs, the degree of cross‐linking was decreased to 0.4% compared to the previously characterized concentration [22].

**FIGURE 1:**
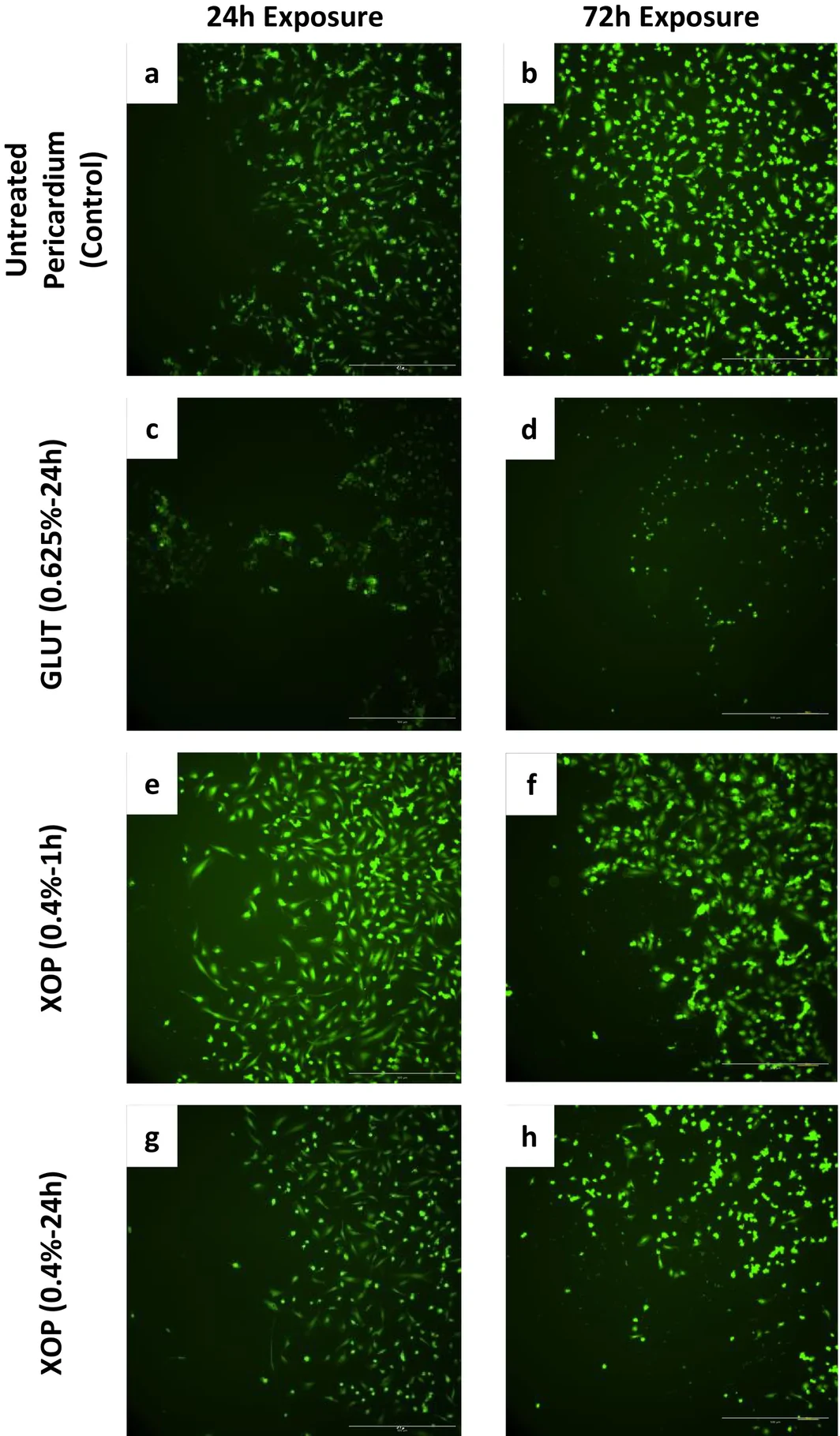
Microscopes (ROIs; 10× magnification) of the cell viability by LIVE/DEAD staining for the untreated (a, b), GLUT‐ (c, d), and XOP‐ (e, f, or g, h) treated groups after 24 h exposure time (left column) according to ISO 10993‐5 [27] and 72 h exposure time (right column). Living cells are shown in green. The scale bar shows a dimension of 500 μm. [Color figure can be viewed at wileyonlinelibrary.com]

The biocompatibility of the optimized treatment method was assessed in vitro by means of a direct contact assay. Since the optimized treatment method has a duration of 1 h, we utilized tissue cross‐linked for 1 h, as well as for 24 h. The evaluation was carried out after an exposure time of 24 h as outlined in the standard [27], and additionally after 72 h (as shown in Figure 1).

The qualitative assessment was conducted utilizing LIVE/DEAD staining. The XOP‐treated groups only show a minor reduction of cell growth and changes in morphology compared to the control group, indicating no to slight cytotoxicity. The GLUT‐treated group shows nearly complete destruction of the cells and many morphologic changes compared to the control group (round in appearance), indicating severe cytotoxicity.

After 1 h of XOP cross‐linking and an exposure time of 24 h, the MTS assay showed cell viability for the untreated control, GLUT (0.625% for 24 h), and XOP (0.4% for 1 h) groups of 100%, 5.1%, and 99.09%, respectively. After an exposure time of 72 h, the cell viability for the untreated control, GLUT (0.625% for 24 h), and XOP (0.4% for 1 h) groups was 100%, 0%, and 90.44%, respectively.

**FIGURE 2:**
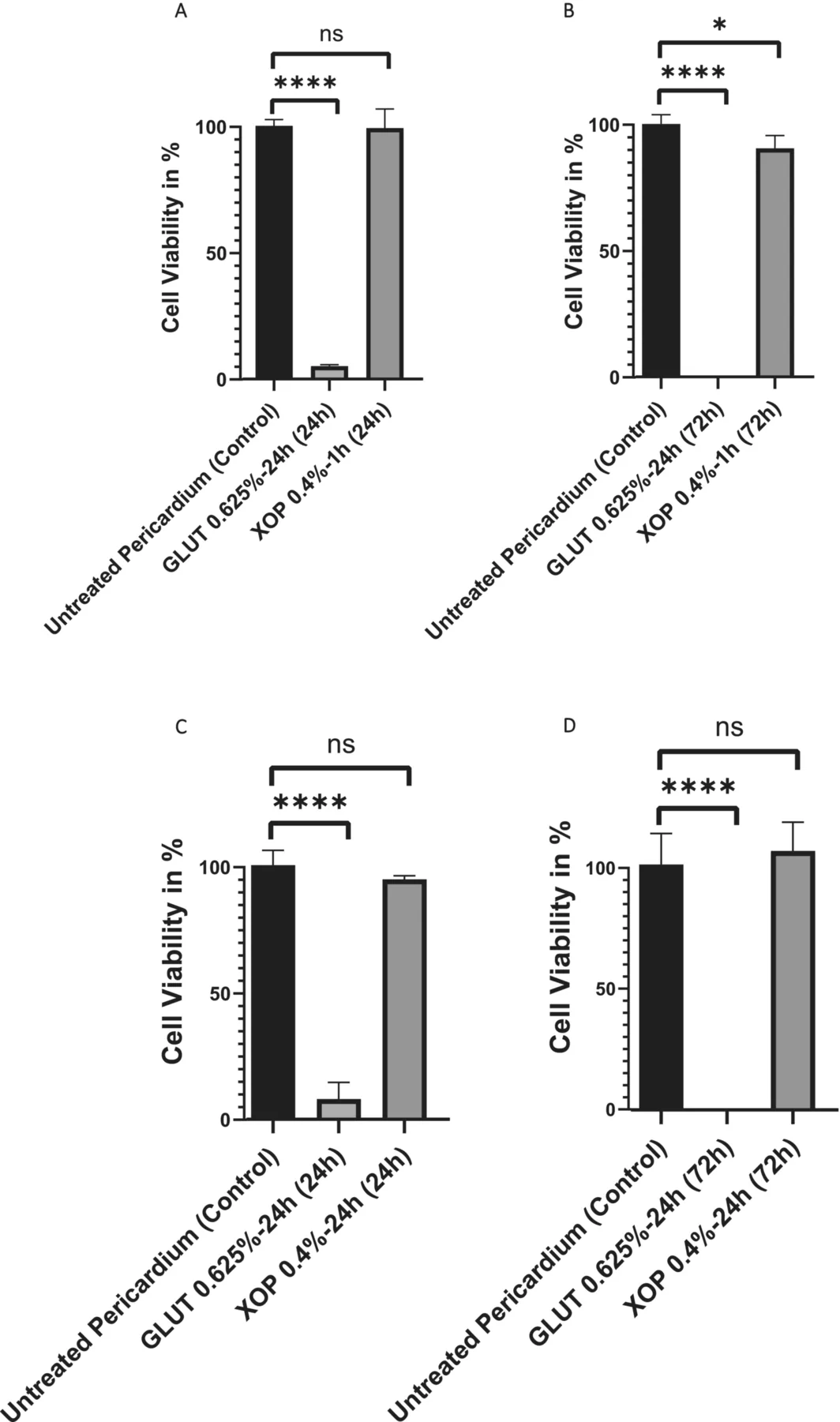
Cell viability assessed by MTS assay after (A) 1 h cross‐linking for XOP and 24 h for GLUT and 24 h exposure time, (B) 1 h cross‐linking for XOP and 24 h for GLUT and 72 h exposure time, (C) 24 h cross‐linking for XOP and 24 h for GLUT and 24 h exposure time (according to ISO 10993‐5 [27]), and (D) 24 h cross‐linking for XOP and 24 h for GLUT and 72 h exposure time, showing a significant and no significant difference between the untreated control and GLUT and XOP treatment, respectively (calculated from raw data). Not significant (ns) = *p* > 0.05, *p ≤ 0.05, and ****p ≤ 0.0001.

After 24 h of cross‐linking and an exposure time of 24 h, cell viability for the untreated control, GLUT (0.625% for 24 h), and XOP (0.4% for 24 h) groups was 100%, 7.82%, and 91.89%, respectively. After an exposure time of 72 h, the cell viability for the untreated control, GLUT (0.625% for 24 h), and XOP (0.4% for 24 h) groups was 100%, 0%, and 100.00%, respectively (as shown in Figure 2).

### 3.2 Comparison of Pericardium and Peritoneum

#### 3.2.1 Collagenase Assay

**FIGURE 3:**
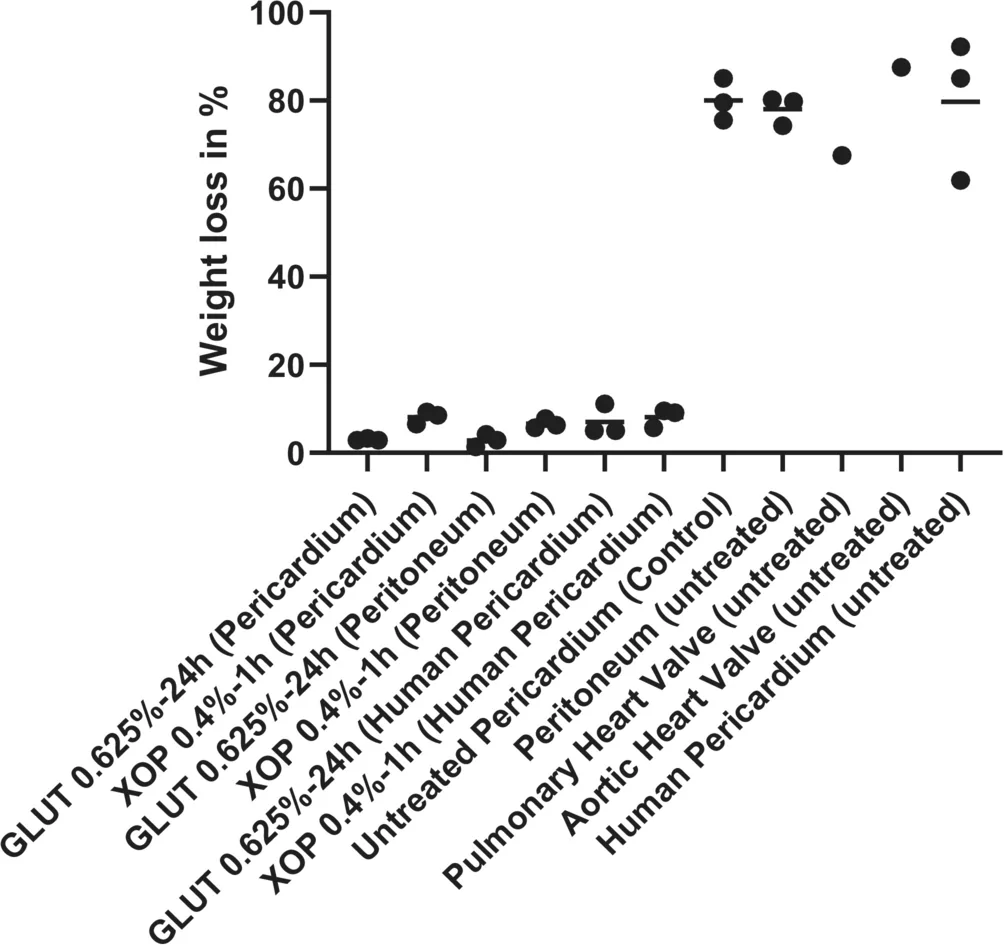
Weight loss of pericardium or peritoneum after GLUT or XOP cross‐linking or untreated pericardium, peritoneum, or heart valves after collagenase treatment. Individual values and the mean of each group (if applicable) are shown.

A collagenase assay on GLUT‐ and XOP‐treated samples was conducted to determine the cross‐linking method's transferability to peritoneum. Furthermore, it was utilized to show if the treatment protects the tissues sufficiently against enzymatic degradation. The pericardium GLUT (0.625% for 24 h), pericardium XOP (0.4% for 1 h), peritoneum GLUT (0.625% for 24 h), peritoneum XOP (0.4% for 1 h), the human pericardium GLUT (0.625% for 24 h), and the human pericardium XOP (0.4% for 1 h) groups showed a weight loss of 3.1%, 8.1%, 2.8%, 6.6%, 7.0%, and 8.1%, respectively. The respective groups showed no significant differences concerning GLUT or XOP treatment (as shown in Figure 3).

Furthermore, the collagenase assay was conducted on untreated samples to assess the collagen content within the different tissues. The (untreated) pericardium, peritoneum, pulmonary heart valve, aortic heart valve, and human pericardium showed a weight loss representing the collagen content of 80.1%, 78.1%, 67.5%, 87.5%, and 79.7%, respectively. The groups showed no significant differences (as shown in Figure 3).

#### 3.2.2 Elastase Assay

**FIGURE 4:**
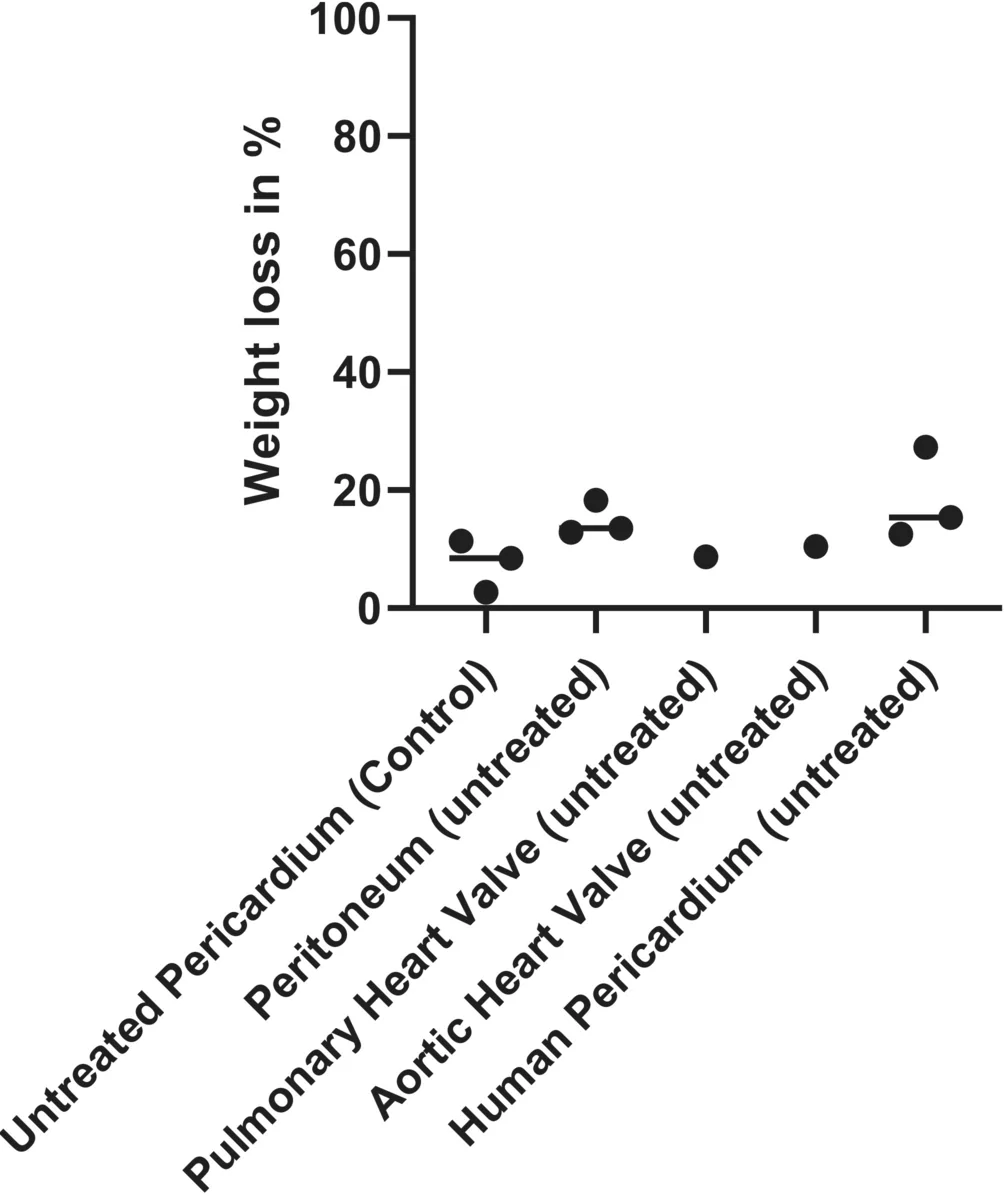
Weight loss of untreated pericardium, peritoneum, or heart valves after elastase treatment. Individual values and the mean of each group (if applicable) are shown.

An elastase assay was conducted on untreated samples to assess the elastin content within the different tissues. The pericardium, peritoneum, pulmonary heart valve, aortic heart valve, and the human pericardium showed a weight loss representing the elastin content of 7.5%, 14.9%, 8.7%, 10.4%, and 18.4%, respectively. The peritoneum showed a higher elastin amount compared to the pericardium (except for the human sample). The groups did not show any significant differences (as shown in Figure 4).

#### 3.2.3 Thermoanalytic Testing

DSC was utilized to assess the denaturation profile of the tissues after treatment with 0.4% XOP for 1 h.

**FIGURE 5:**
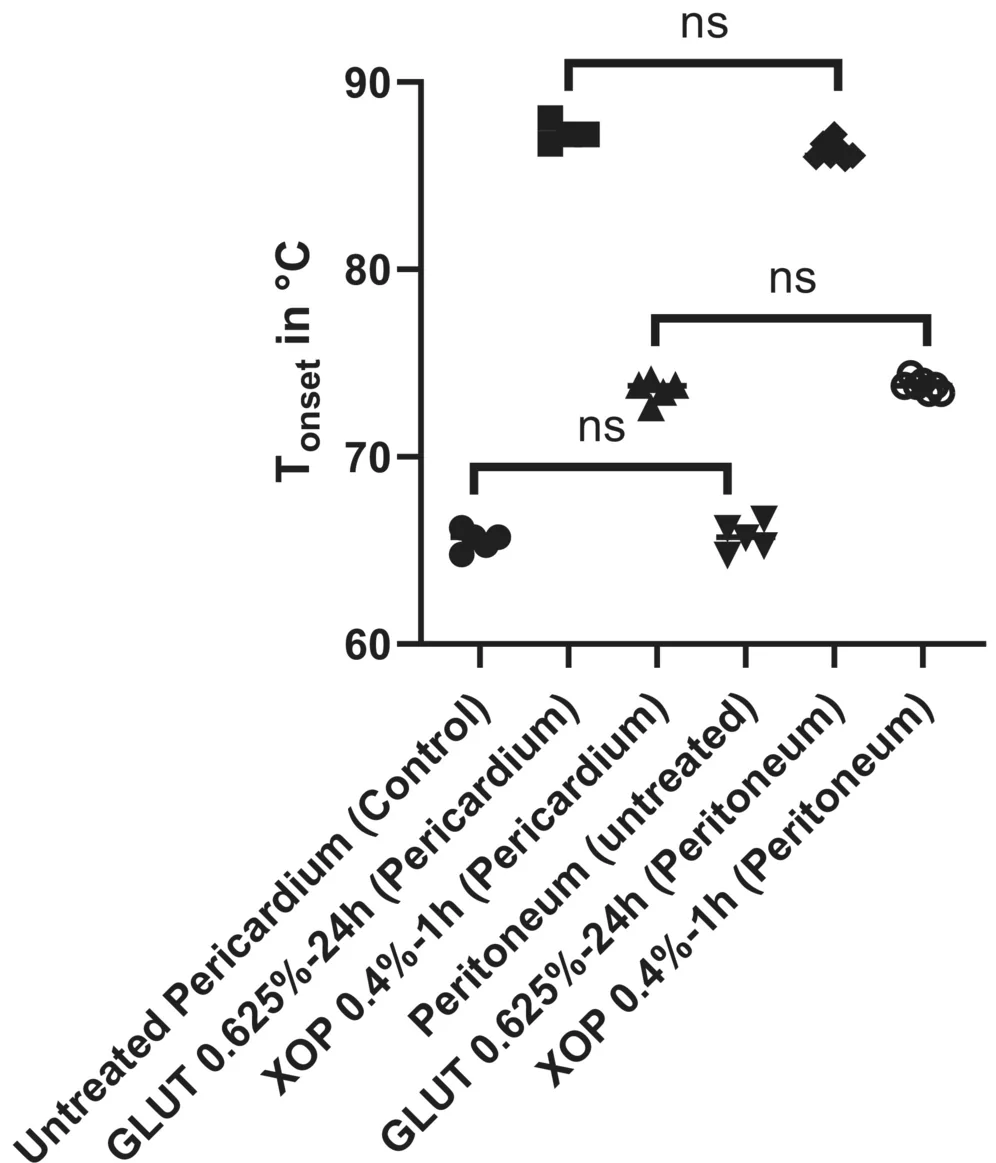
*T*_onset_ of pericardium and peritoneum, untreated or treated with GLUT or XOP. Not significant (ns) = *p* > 0.05.

The untreated pericardium, pericardium GLUT (0.625% for 24 h), pericardium XOP (0.4% for 1 h), untreated peritoneum, peritoneum GLUT (0.625% for 24 h), and peritoneum XOP (0.4% for 1 h) groups showed an average *T*_onset_ of 65.5°C ± 0.7°C, 87.3°C ± 0.6°C, 73.5°C ± 0.7°C, 65.7°C ± 0.9°C, 86.4°C ± 0.8°C, 73.8°C ± 0.6°C, respectively. The different tissues with the same treatment showed no significant differences (as shown in Figure 5).

#### 3.2.4 Biomechanical Testing

The young's modulus of the collagen phase (coll‐e), failure strain, and ultimate tensile strength (UTS), representing the tissue's elasticity, maximum strain at failure, and maximum stress at failure, respectively, were assessed by uniaxial tensile testing. A summary of the tissue's biomechanical properties is shown in Table 3.

**TABLE 3:** Biomechanical properties of the different tissues.

Tissue	Treatment	Coll‐e [MPa]	Failure strain [mm/mm]	UTS [MPa]
Pericardium (porcine)	Untreated	57.64 ± 4.33	0.35 ± 0.07	12.41 ± 1.45
	GLUT 0.625%, 24 h	37.19 ± 12.68	0.91 ± 0.23	10.43 ± 0.33
	XOP 0.4%, 1 h	76.51 ± 15.09	0.62 ± 0.13	19.10 ± 4.95
Peritoneum (porcine)	Untreated	70.58 ± 22.52	1.63 ± 0.29	31.18 ± 9.28
	GLUT 0.625%, 24 h	69.51 ± 15.25	1.21 ± 0.28	24.94 ± 4.91
	XOP 0.4%, 1 h	77.66 ± 23.94	1.43 ± 0.29	31.51 ± 8.42
Pericardium (human)	Untreated (*n* = 1)	31.49	0.52	6.48
	XOP 0.4%, 1 h	106.9 ± 47.47	0.70 ± 0.35	30.63 ± 10.72
Peritoneum (human)	XOP 0.4%, 1 h	26.95 ± 11.10	0.45 ± 0.07	6.75 ± 3.46
Heart valve (porcine)	Untreated	43.34 ± 4.80	1.14 ± 0.22	13.13 ± 1.29

**FIGURE 6:**
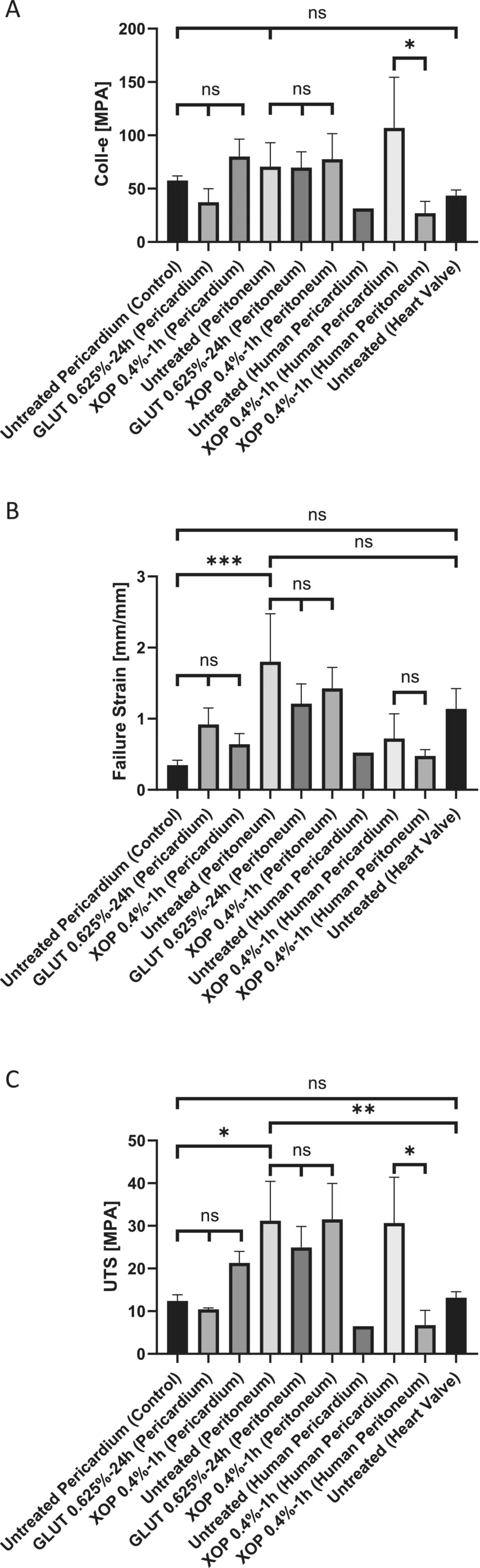
(A) Shows the collagen slope, (B) shows the failure strain, and (C) shows the ultimate tensile strength of the different tissues, untreated or treated with GLUT or XOP. Not significant (ns) = *p* > 0.05, *p ≤ 0.05, **p ≤ 0.01, ***p ≤ 0.001.

The collagen phase represents the load‐bearing capacity of the collagen network, which shows no significant differences within most groups. However, human pericardium and human peritoneum showed a small difference (as shown in Figure 6A).

The failure strain indicates how much a material can be stretched until it breaks, showing no significant difference within most groups. However, the untreated pericardium and the untreated peritoneum showed a significant difference (as shown in Figure 6B).

The ultimate tensile strength provides information about the maximum tension of a tissue until it tears. The samples showed no significant difference within most groups. However, the untreated peritoneum showed differences from the untreated pericardium and heart valve tissue. Furthermore, the human pericardium and human peritoneum showed a small difference (as shown in Figure 6C).

The XOP cross‐linked pericardium shows a higher collagen slope and UTS than the GLUT cross‐linked pericardium.

**FIGURE 7:**
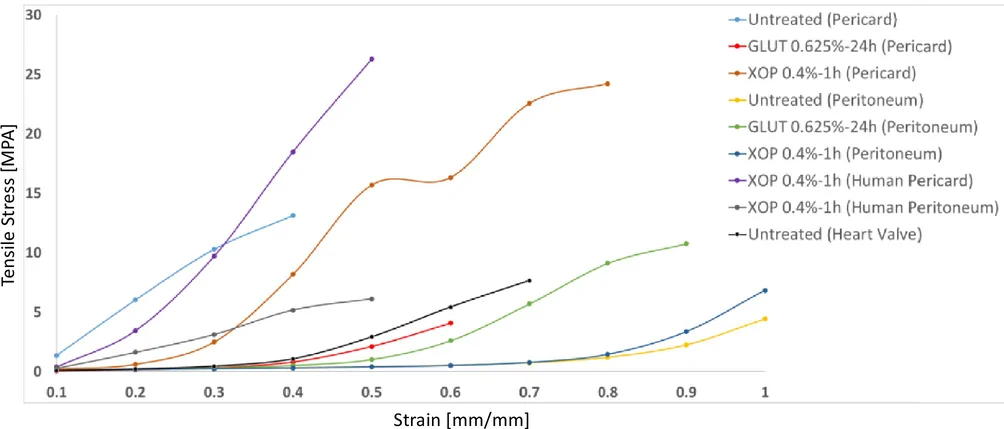
Stress–strain curve of the different tissues, untreated or treated with GLUT or XOP. Results are only shown until a strain of 1.0 mm. [Color figure can be viewed at wileyonlinelibrary.com]

All samples showed a J‐shape stress–strain curve, which is representative of soft tissues [38] (as shown in Figure 7).

The curves indicate that the non‐crosslinked pericardium exhibits a rigid behavior, with increasing extensibility as the cross‐linking degree rises. In contrast, the peritoneum demonstrates high compliance in its uncrosslinked state, becoming stiffer as the cross‐linking degree increases. Heart valve tissue also shows high elasticity, but is located between the two non‐crosslinked tissues. After crosslinking, the mechanical behavior of the tissues becomes more similar to that of the heart valve.

#### 3.2.5 Histology

**FIGURE 8:**
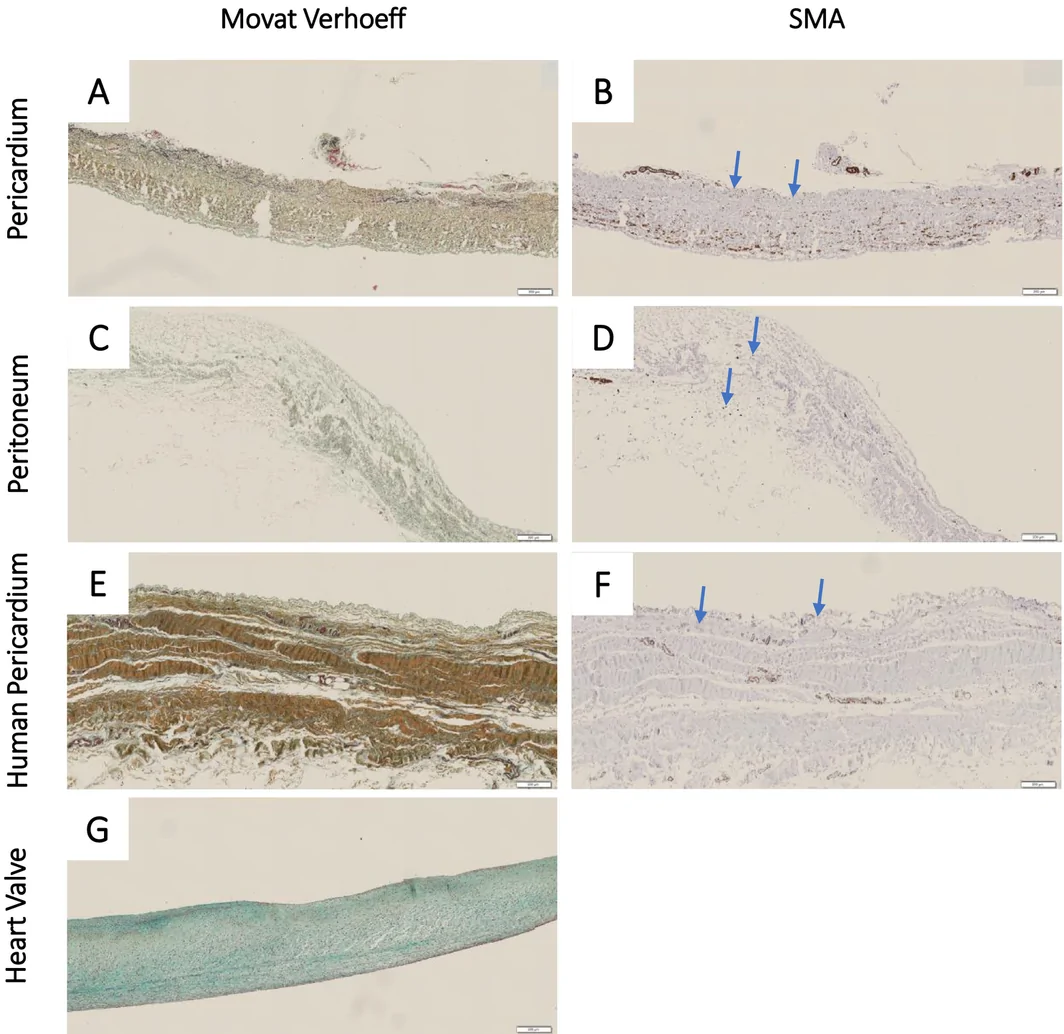
Histological images (ROIs) of untreated porcine pericardium (A, B), and peritoneum (C, D), human pericardium (E, F), and porcine heart valve (G) tissue patches. The left and right column shows the samples stained with Movat Verhoeff or SMA dye, respectively. The blue arrows show exemplary SMA‐colored cells. The scale bar shows a dimension of 200 μm. [Color figure can be viewed at wileyonlinelibrary.com]

Figure 8 shows the composition of the different tissues. Staining the tissue samples with Movat Verhoeff dye enabled the identification of collagen and elastin fibers, whereas smooth muscle actin (SMA) dye allowed the visualization of SMA cells within the tissue. In the native porcine pericardium, densely packed collagen fibers with scattered gaps are observed. Elastin fibers are interspersed among the collagen fibers and are present across all layers, with a significant accumulation in the fibrosa (Figure 8A).

Additionally, interstitial cells are distributed throughout all layers of the pericardium, with pronounced proliferation in the serosa. These cells exhibit a round‐to‐oval shape and are spread uniformly across the tissue. The porcine pericardium shows a high cell density (Figure 8B).

In contrast, the native porcine peritoneum exhibits a loose collagen structure with numerous gaps between fibers. Elastin fibers are evenly distributed across the tissue, without any specific layer showing a concentrated presence. Notably, the porcine peritoneum contains a higher amount of elastin fibers compared to the pericardium (Figure 8C).

Similar to the pericardium, cells in the porcine peritoneum are dispersed across the tissue. These cells generally display a round‐to‐oval shape, although spindle‐shaped structures can occasionally be observed. Although the cells are uniformly distributed, their density is lower than in the porcine pericardium (Figure 8D).

The human pericardium (excised from an elderly patient), on the other hand, contains densely packed collagen with conspicuously thick individual fibers. Towards the outer regions, the tissue becomes looser and interspersed with elastin fibers (Figure 8E).

The cells in the human pericardium have a round‐to‐oval morphology and are evenly distributed throughout the tissue, with the majority located in the serosa. Like the porcine counterpart, the human pericardium contains a high number of cells (Figure 8F).

The porcine heart valve tissue exhibits densely packed collagen across all layers. Elastin is the most abundant in the lamina ventricularis. Some gaps are present in the central region of the tissue. Cells are distributed throughout all layers, with the lamina fibrosa showing the highest cell density. The typical tri‐layer structure could not be observed (Figure 8G).

All tissues show similar ECM components. However, they differ in the number of single structures. The peritoneum's collagen and elastin distribution is comparable to that of the pericardium. Furthermore, the packing of the tissues varies greatly. The heart valve is the most densely packed, followed by the densely packed pericardium. The peritoneum represents rather loose connective tissue. The pericardium shares similarities with the heart valve tissue regarding ECM composition and packaging, whereas the peritoneum shows a similar cell morphology.

#### 3.2.6 Fiber Alignment

**FIGURE 9:**
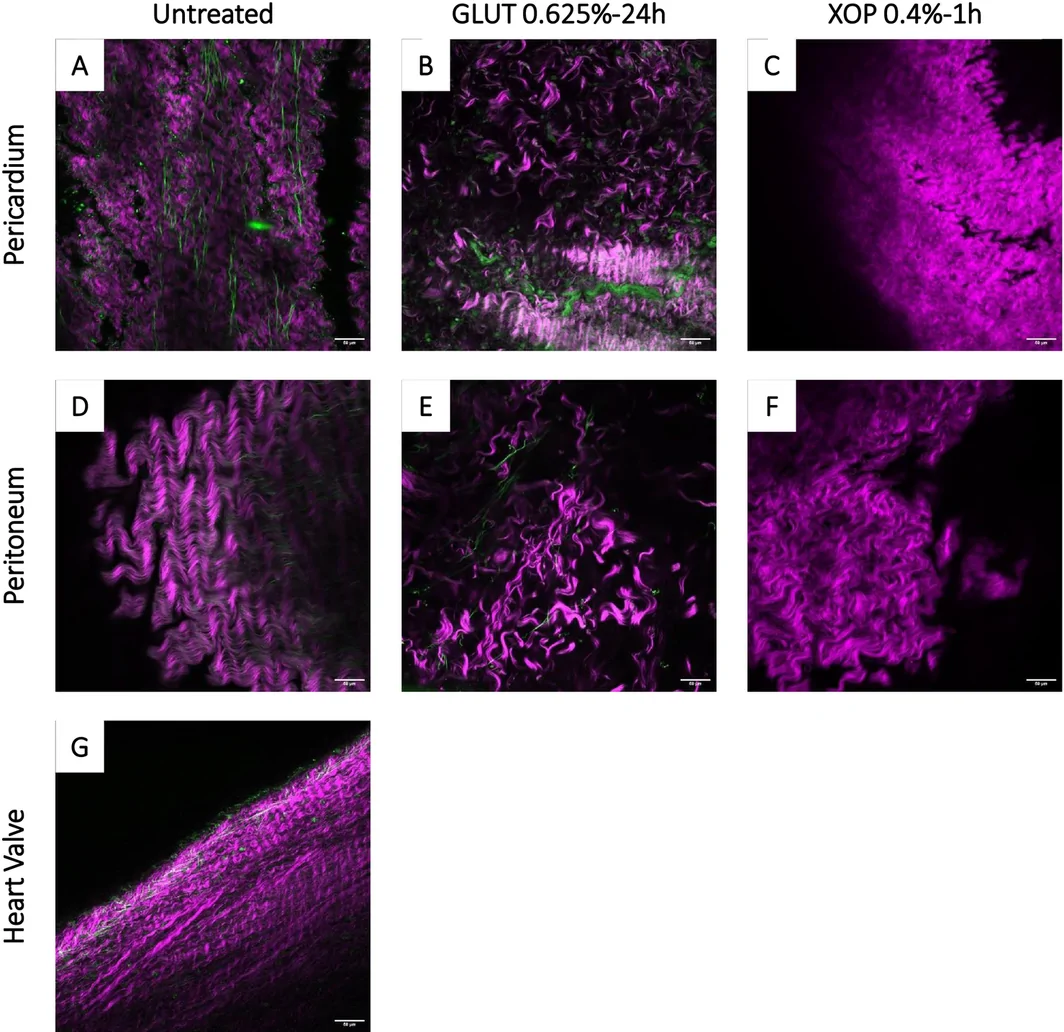
Overlay images (ROIs) of the SHG signal (collagen, shown in purple) and the autofluorescence signal (elastin, shown in green) of the pericardium (A–C), peritoneum (D–F), and heart valve tissue (G). The left, middle, or right column, shows the tissue untreated, GLUT‐or XOP‐treated, respectively. Due to the strong signal overlap of the channels after cross‐linking in the XOP group, only the collagen channel is shown. The scale bar shows a dimension of 50 μm. [Color figure can be viewed at wileyonlinelibrary.com]

Two‐photon imaging was performed to visualize the fiber alignment of the tissue in its native state as well as after treatment (as shown in Figure 9). The SHG and autofluorescence signals were used to visualize collagen and elastin, respectively. Both channels appear to overlap after GLUT‐ and XOP‐treatment, which was described as the fusing of the collagen and elastin fluorescence signals due to the formation of additional cross‐links [39, 40].

The untreated heart valve exhibits the most densely packed collagen fibers regarding collagen fiber windings but also regarding the gaps between the fibers, followed by the pericardium and the peritoneum, displaying the loosest arrangement. All tissues show elastin fibers between the collagens. After GLUT‐ and XOP‐treatment, the index of fiber alignment appears to be altered, although it is more pronounced after GLUT cross‐linking. In addition, the distances between the fibers are significantly increased.

#### 3.2.7 Hydrodynamic Testing

**FIGURE 10:**
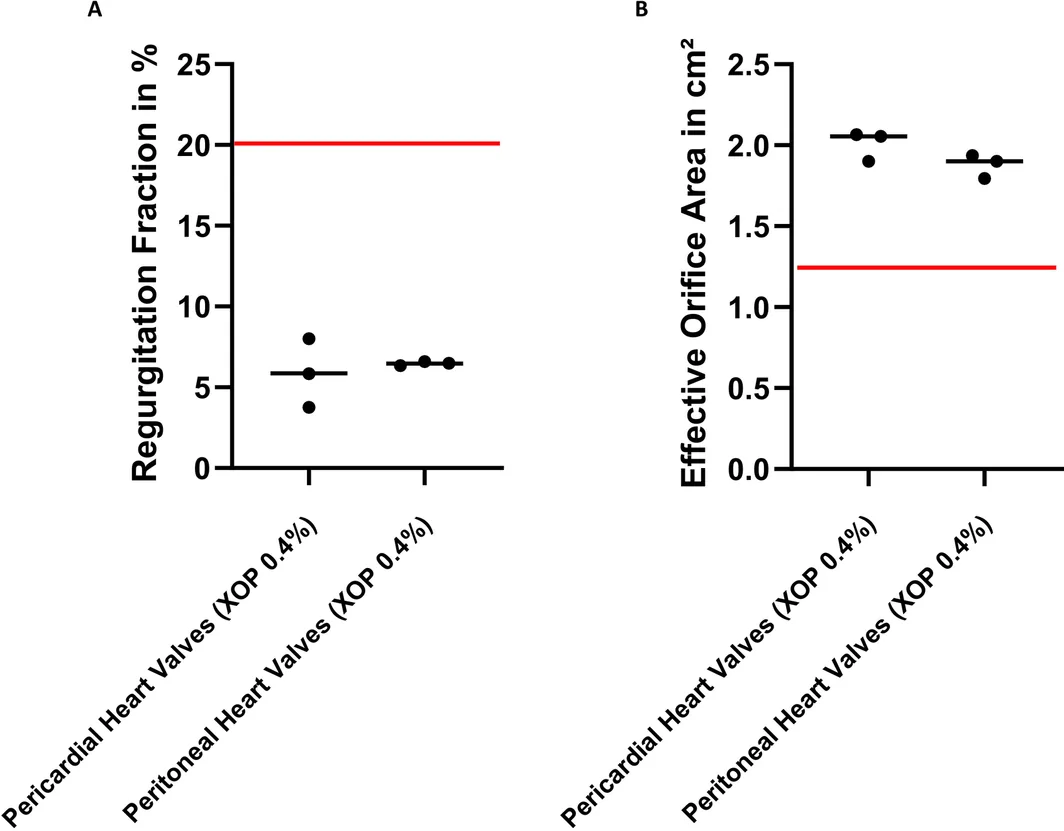
(A) Shows the RF of the pericardial and peritoneal heart valve constructs. The maximum RF of 20% (red line) specified in the standard [37] was not exceeded by any heart valve construct. (B) Shows the EOA of the pericardial and peritoneal heart valve constructs. None of the heart valve constructs tested fell below the minimum standard of 1.25 cm^2^ (for heart valves with a respective valve diameter, red line) specified in the guidelines [37]. Individual values and the mean of each group are shown. [Color figure can be viewed at wileyonlinelibrary.com]

Acute hydrodynamic testing was performed to evaluate the short‐term mechanical performance of pericardial and peritoneal heart valve constructs treated with 0.4% XOP. Therefore, the EOA and the RF were recorded, representing the heart valve construct's opening‐ and closing‐ behavior, respectively. The inner diameter of each scaffold was 22 mm. The average RF and EOA of the pericardial heart valve constructs were 5.9% and 2.0 cm^2^, respectively. The average RF and EOA of the peritoneal heart valve constructs were 6.5% and 1.9 cm^2^, respectively (as shown in Figure 10). Both tissues exhibited similar short‐term performance and remained within the standard threshold [37].

## 4 Discussion

This work was conducted to identify alternative tissue sources to the clinically established pericardium and to optimize a treatment method for the intraoperative manufacturing of autologous heart valve constructs. Tissue treatment via cross‐linking is essential for manufacturing a prosthesis from biological tissues, as it enhances the material's mechanical properties and offers protection against enzymatic degradation [2, 23, 41]. However, since the rate of tissue degeneration is proportional to the amount of non‐living tissue [12], the cytotoxic profile of the commercially utilized GLUT is a primary factor contributing to the long‐term failure of current heart valve prostheses [26]. Therefore, identifying a less‐cytotoxic cross‐linking agent for the treatment of biological heart valve material is crucial.

Our cytocompatibility evaluation shows that only GLUT‐treated samples show a significant cytotoxic effect, according to the standard [27]. For the quantitative cytotoxicity evaluation, a reduction of the cell viability to less than 20% is considered a cytotoxic effect [27], which is only true for the GLUT‐treated samples. The cytotoxicity of GLUT utilizing MTT assay has also been reported in different cell types elsewhere [13, 42]. XOP‐treated tissue exhibits a significantly higher cytocompatibility, making it a superior alternative to GLUT.

Furthermore, the antigenicity of xenogeneic heart valve prostheses has been identified as one major problem of calcification, especially in pediatric and young patients [43], whereas heart valves from autologous pericardial tissue indicated superior biocompatibility in pediatric patients compared to xenogeneic ones [35]. Comparable biocompatibility was also described for peritoneum [44].

Therefore, the combination of XOP treatment with autologous tissue may represent a promising approach for generating biocompatible valve constructs with potential for further investigation.

Tissue treated with GLUT or XOP showed significantly higher resistance to collagenase compared to untreated samples. However, the resistance of GLUT‐treated samples was higher than XOP‐treated ones, showing less weight loss and therefore a higher cross‐linking degree. Although protection against enzymatic degradation is crucial, excessive resistance may also have detrimental effects. Metalloproteinases like collagenase are important remodeling enzymes [45, 46] that must retain their ability to degrade and therefore facilitate the production of new tissue (remodeling). However, excessive cross‐linking inhibits M2 macrophage polarization and limits cell infiltration [47], both crucial for a potential regenerative heart valve. Therefore, the slightly higher affinity of XOP‐treated tissue towards metalloproteinases may be advantageous for positive remodeling of the heart valve. The similar weight loss between pericardium and peritoneum treated with XOP indicates a similar cross‐linking degree after treatment. However, elsewhere peritoneum has been reported to exhibit superior resistance to enzymatic degradation compared to the pericardium [44]. The extent to which the balance between enzymatic stability and degradability translates into functional remodeling in a three‐dimensional construct remains to be investigated.

The biochemical assays on untreated samples indicated a similar amount of collagen between both tissues, which is similar to the results of other groups [29, 48]. The elastin content in peritoneum was shown to be higher compared to pericardium, which was also described before [44]. However, the human pericardial tissue showed an even higher elastin amount, which could be influenced by the individual's age [49]. Moreover, the untreated samples showed no significant differences to GLUT‐ or XOP‐treated samples after elastase treatment (data not shown), indicating no significant elastin cross‐linking effect of the reagents, which is congruent to findings described elsewhere [29]. Since elastin, which plays a crucial role in heart valve mechanics (elastic recoil), remains uncross‐linked, it is particularly susceptible to degradation [8, 50]. As elastin is only barely re‐synthesized during lifetime [51], the initial higher elastin content of the peritoneum may be advantageous for elastic recoil; however, its impact on long‐term mechanical stability under cyclic loading remains unclear.

The similar degree of cross‐linking within both tissues after XOP treatment was confirmed by the DSC measurements. With regard to the initial *T*_onset_ of the untreated tissues, the temperature of both tissue types after treatment indicates an identical amount of newly inserted cross‐links. Therefore, the identified treatment of 0.4% XOP for 1 h of pericardium can be evaluated as transferable to the peritoneum regarding the cross‐linking degree.

The higher elastin content of the peritoneum was also reflected in the tensile test results. The tissue shows a larger elastin phase compared to the pericardium, as defined elsewhere [38], requiring less stress for a corresponding strain. This was confirmed by the higher failure strain and UTS of the peritoneum, which has been described elsewhere as an advantage over pericardium when used as a cardiovascular material [44]. Interestingly, cross‐linking of the tissues has the opposite effect on their mechanical performances. Although the pericardium becomes more elastic, the peritoneum becomes stiffer as the degree of cross‐linking increases (untreated > XOP > GLUT), which is concurrent with our former results [22]. This trend is confirmed by the increasing or decreasing failure strain of the pericardium or peritoneum, respectively. The heart valve tissue is more elastic than pericardium and, in terms of its mechanical properties, resembles the peritoneum (albeit not quite as elastic). This observation is consistent with the elastin content, which is also located between both tissue types.

Furthermore, the stress–strain curves of the peritoneum show that the collagen phase becomes more prominent after cross‐linking, indicating increased or earlier stress‐bearing of the collagen fibers. However, these mechanical differences should not be interpreted as indicative of suitability for longevity, as cyclic long‐term durability and creep behavior were not assessed in this study. Although research indicates that small differences in cross‐linking degrees have minimal impact on the mechanical properties of human pericardium [52], tissues treated with GLUT and XOP exhibit noticeably distinct properties after treatment. Since the mechanical characteristics of soft tissues are influenced by their microstructure [53], the different effects of cross‐linking are likely attributable to their initial ECM structure. As supportively shown by the histology, pericardium is primarily composed of dense [54], and peritoneum of loose [55] connective tissue, respectively. Since collagen provides a high cell adhesive surface [56], the ECM gaps within the peritoneum could facilitate cell attachment. Depending on the type of colonizing cells, this could be advantages or disadvantages. For instance, colonization by M1 macrophages could be unfavorable, as they are associated with pro‐inflammatory responses, whereas colonization by M2 macrophages could be favorable, given their primary role in anti‐inflammatory responses [57]. However, the extent of cell infiltration into the full three‐dimensional structure was not assessed in this study.

The structure of the peritoneum leads to high collagen modulus values despite its overall elastic behavior. This suggests that the tissue maintains a pronounced toe region, allowing initial extensibility, whereas the collagen network provides strong resistance once the fibers are fully recruited, which could be beneficial regarding mechanical behavior. However, a loose tissue structure is currently assumed to be detrimental to long‐term durability.

After cross‐linking, the collagen fibers in both tissues are affected, leading to a decreased orientation index, as described elsewhere [34]. The proportion of decreasing fiber alignment and increasing cross‐linking degree can also be seen in the 2‐photon images.

The data suggest that in pericardium, the changes in fiber alignment are the key factor influencing its mechanical behavior after cross‐linking. If stress is exerted on the cross‐linked tissue, the more isotropic structure exerts less shear forces inter‐fibrillary [58], leading to less constraint of the tissue along specific axes. In Pericardium, chemical cross‐linking increases local stiffness but enhances global elasticity. In addition to the lower elastin content of pericardium, the denser fiber packing also contributes to its higher stiffness [59] compared to peritoneum.

In contrast, the mechanical behavior of peritoneum is more influenced by the formation of additional cross‐links between adjacent collagen molecules. The initial loose state of its ECM allows the fibers to slide past each other easily. After cross‐linking the fibers, they experience high shear forces under applied stress, resulting in increased tissue stiffness. Furthermore, cross‐linking leads to tissue retraction [60], which could further increase the shear forces within peritoneum. Although both tissues begin to show biomechanical characteristics that partially overlap with those of native heart valve tissue after cross‐linking, this occurs at the expense of an altered structure.

Despite the tissue's initial differences, no differences were observed under the applied acute testing conditions. However, the present study does not address long‐term fatigue behavior or creep, as hydrodynamic testing was limited to acute conditions over a small number of cycles. The increased compliance and higher failure strain observed in peritoneal tissue may raise concerns regarding its dimensional stability under physiological cyclic loading. In particular, excessive tissue stretching could impair leaflet coaptation and lead to regurgitation over time. Therefore, accelerated wear testing will be essential to evaluate the long‐term mechanical performance of peritoneum‐based constructs.

For long‐term functional valve performance, the ECM structure of a heart valve must not only be mimicked but also possess the ability to undergo adaptive remodeling, a process driven by mechanosensitive cells [8, 11]. Although the regenerative capacity of heart valves converted from pericardium and peritoneum only becomes evident over time in vivo, their similar cell population may be favorable. However, the capacity for functional remodeling cannot be inferred from the present data.

In summary, structurally, the pericardium shows more similarities to a heart valve, whereas the peritoneum exhibits distinct biomechanical properties. Further research is needed to determine which factor is most critical for the development of a durable heart valve construct.

## 5 Conclusion

The limitations of current heart valve prostheses may be partially addressed by the use of autologous tissues in combination with appropriate treatment methods. In this study, a cross‐linking approach was optimized to enable intraoperative tissue treatment. Furthermore, peritoneum was investigated as a potential alternative to the clinically established pericardium. The results demonstrate that both tissues share comparable extracellular matrix components, whereas exhibiting distinct structural and biomechanical characteristics. In particular, the higher elastin content and looser organization of peritoneum result in increased compliance compared to pericardium.

The feasibility of shaping both tissues into valve‐like constructs and their adequate acute hydrodynamic performance under standardized in vitro conditions was demonstrated. However, the observed mechanical differences, especially the increased extensibility of peritoneum, may influence its behavior under long‐term physiological cyclic loading.

Overall, peritoneum represents a potential autologous tissue candidate for further investigation in the context of intraoperative heart valve construct manufacturing. Nevertheless, its long‐term mechanical stability, resistance to creep, and impact on valve function remain to be determined. Therefore, the present results should be interpreted as an initial feasibility assessment rather than evidence of long‐term functional suitability.

## 6 Outlook

Further studies are required to evaluate the suitability of peritoneum for sustained valve function under physiological conditions. In particular, accelerated wear testing and creep analysis will be essential to assess potential long‐term deformation and its impact on leaflet coaptation and regurgitation. In addition, a comprehensive biological evaluation is necessary, including investigation of cellular infiltration into the full three‐dimensional structure and the capacity for extracellular matrix production, which is critical for maintaining valve functionality over time. Future in vivo studies will be required to assess remodeling behavior, calcification potential, and long‐term performance in a physiological environment. Moreover, the translation of findings from porcine to human tissue must be further validated.

## 7 Limitations

This study has several limitations. First, hydrodynamic testing was limited to acute conditions and a small number of cycles, which does not allow assessment of long‐term durability, fatigue behavior, or creep. Second, while cytocompatibility was demonstrated, the study does not evaluate cellular infiltration into the full three‐dimensional structure or the long‐term production of extracellular matrix. Third, sample sizes for human tissues were limited. Finally, no in vivo validation was performed, which will be necessary to assess remodeling capacity, calcification, and long‐term functionality.

## Author Contributions

Marvin Steitz: concept/design, data collection, data analysis/interpretation, drafting article. Mahamuda Badhon Khan: data collection, data analysis, revision of article. Katharina Fritsch: data analysis, revision of article. Alexander Breitenstein‐Attach: data collection, data analysis, revision of article. Jana Katharina Betty Korbacher: data collection. Jordi Joaquin Modolell Y Mainou: data collection. Katrin Schrimpf: data collection. Robert Ramm: data collection. Sara Knigge: data collection, data analysis. Katja Eildermann: data collection, data analysis. Georg Arlt: data collection, revision of article. Antonia Schulz: revision of article. Jonathan Frederik Sebastian Kiekenap: data collection. Boris Warnack: revision of article. Peter Kramer: data collection, revision of article. Frank Edelmann: revision of article. Felix Berger: revision of article. Boris Schmitt: data collection, data analysis, revision of article.

## Conflicts of Interest

The authors Marvin Steitz, Mahamuda Badhon Khan, Alexander Breitenstein‐Attach, Jordi Joaquin Modolell Y. Mainou, Boris Warnack, and Boris Schmitt are (partially) employed by GrOwnValve Ltd.

## Data Availability

The data that support the findings of this study are available on request from the corresponding author. The data are not publicly available due to privacy or ethical restrictions.
